# Citrulline deiminase pathway provides ATP and boosts growth of *Clostridium carboxidivorans* P7

**DOI:** 10.1186/s13068-021-02051-4

**Published:** 2021-10-16

**Authors:** Xiangfei Li, Rumeng Han, Teng Bao, Tolbert Osire, Xian Zhang, Meijuan Xu, Taowei Yang, Zhiming Rao

**Affiliations:** 1 The Key Laboratory of Industrial Biotechnology, Ministry of Education, School of Biotechnology, Jiangnan University, 1800 Lihu Road, Wuxi, 214122 Jiangsu China; 2 Department of Chemical and Biomolecular Engineering, The Ohio State University, Columbus, OH 43210 USA

**Keywords:** *Clostridium carboxidivorans*, Citrulline, Syngas fermentation, Alcohol/acid ratio

## Abstract

**Background:**

*Clostridium carboxidivorans* P7 is capable of producing ethanol and butanol from inexpensive and non-food feedstock, such as syngas. Achieving improved ethanol and butanol production in the strain for industrial application depends on the energetics and biomass, especially ATP availability.

**Results:**

This study found that exogenous addition of citrulline promoted accumulation of ATP, increased specific growth rate, and reduced the doubling time of *C. carboxidivorans* P7. In heterotrophic fermentation experiments, the addition of citrulline increased intracellular ATP by 3.39-fold, significantly enhancing the production of total alcohol (ethanol + butanol) by 20%. Moreover, in the syngas fermentation experiments, the addition of citrulline improved the level of intracellular ATP and the biomass by 80.5% and 31.6%, respectively, resulting in an 18.6% and 60.3% increase in ethanol and the alcohol/acid production ratio, respectively.

**Conclusions:**

This is the first report that citrulline could promote the growth of *C. carboxidivorans* P7 and increase the level of intracellular ATP, which is of great significance for the use of *C. carboxidivorans* P7 to synthesize biofuels.

## Background

Globally, the main energy source for human survival is petroleum-based non-renewable fossil fuels [1]. Nowadays, fossil fuel depletion, increasing energy consumption, growing CO_2_ emissions, and climate change have increased the demand for renewable energy sources [2, 3]. As renewable biomass fuels, ethanol [4] and butanol [5] have high energy density and are compatible with current petroleum energy infrastructure equipment, which has attracted significant attention [6]. However, the production of bio-butanol and bio-ethanol is presently limited owing to the high substrate cost of conventional feedstocks, such as starch and molasses [7]. In recent years, gas fermentation has attracted increasing attention as an emerging method for the production of renewable biofuels, such as bio-butanol and bio-ethanol [8, 9]. On one hand, the feedstocks for gas fermentation are relatively abundant and inexpensive, such as exhaust gas from industrial production, and burning fossil fuels, as well as synthetic gas. On the other hand, compared with traditional acetone–butanol–ethanol (ABE) fermentation by solventogenic Clostridium, gas fermentation does not occupy food resources and precious land resources of humans [10–13]. However, low solubility of gaseous substrates in water, low ATP generation, and poor biomass of solventogenic Clostridium are still the major factors limited the production of bio-butanol and bio-ethanol [14, 15].

*Clostridium carboxidivorans* P7 is one of the strains capable of producing biofuel from syngas [16]. It can synthesize ethanol and n-butanol by capturing CO_2_, CO, and H_2_ through WLP (Wood–Ljungdahl pathway) [17, 18]. However, whole-genome sequencing (GenBank: CP011803.1) indicates that there is an incomplete TCA cycle in *C. carboxidivorans* P7 [16]. Hence, its own energy supply mainly comes from the glycolytic pathway and the synthetic pathway for acetic acid production. What is more, syngas fermentation also has drawbacks, such as low biomass and insufficient energy supply, resulting in low biofuel production and increased by-products accumulation. Although optimization of the appropriate media composition, metal ion composition [19], pH [20, 21], and reactor design [22] can improve cell growth and product accumulation, it is not of help in increasing intracellular ATP production. Therefore, there is an urgent need to enhance intracellular ATP supply and biomass of *C. carboxidivorans* P7, so as to promote the biofuels production. Nowadays, genetic engineering is regarded as a useful strategy to manipulate the metabolism and to enhance accumulation of ideal products [23–25]. However, the molecular genetic manipulation technology of *C. carboxidivorans* P7 had just been established [7], it required relatively long operation time, and the success rate was not high. So, investigators have noted that amino acids, as the basic building blocks of proteins, play a crucial role in the life of cells [26]. It is known that arginine can be hydrolyzed by the action of arginase to produce urea and ornithine. The ornithine could react with carbamoyl phosphate to generate citrulline by ornithine carbamoyltransferase. Finally, citrulline can be transformed to carbon dioxide, water, ammonia, and two molecules of ATP by carbamoyl phosphate synthetase [27]. The addition of arginine shortened the *Clostridium autoethanogenum* doubling time, increased the intracellular ATP energy level by fivefold, and weakened the accumulation of by-product acetic acid [15]. Therefore, amino acid metabolism has an enormous potential to improve the cell density and intracellular energy level of *C. carboxidivorans* P7.

This paper assessed the significance of amino acids which could be helpful to promote the biomass, ATP regeneration, and the yield of biofuels. It was found that citrulline could provide additional ATP and enhance biomass and specific growth rate for *C. carboxidivorans* P7 under both heterotrophic and autotrophic conditions. What is more, the addition of citrulline increased the alcohol/acid production ratio by 60.3% under autotrophic conditions. The results provided a new method of thinking for promoting cell growth and increasing energy levels in *C. carboxidivorans* P7.

## Results

### Citrulline boosted cell growth under heterotrophic conditions

**Fig. 1:**
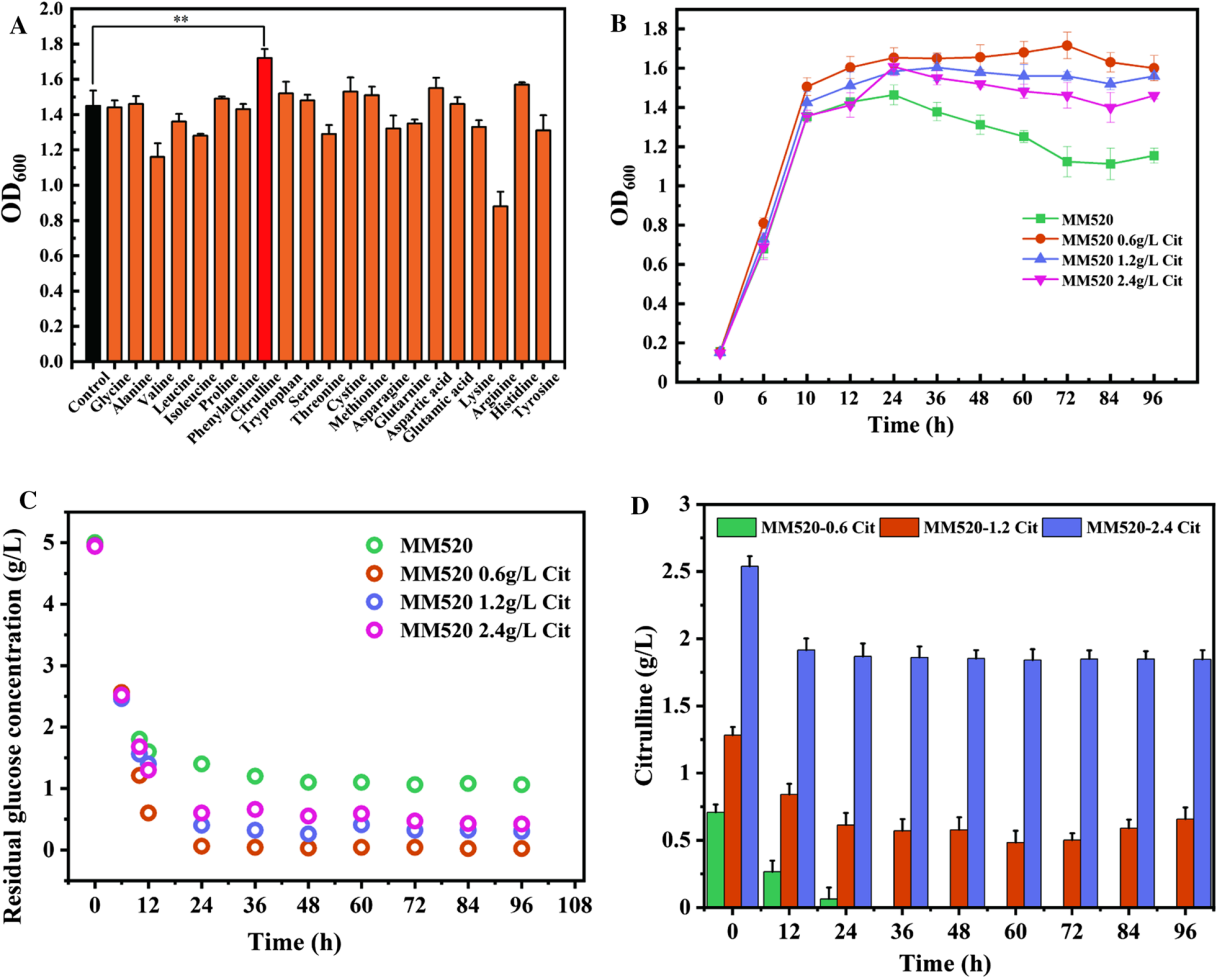
Effects of different of amino acids and different concentration of citrulline on the growth of *C. carboxidivorans* P7. **A** The effects of 21 amino acids on the growth of *C. carboxidivorans* P7. **B** Effects of different concentrations of citrulline on the growth of *C. carboxidivorans* P7. **C** Effects of different concentrations of citrulline on sugar consumption of *C. carboxidivorans* P7. **D** The concentration of residual citrulline under the addition of 0.6 g/L, 1.2 g/L, or 2.4 g/L citrulline. **P < 0.05

Amino acids, as the basic units of proteins, play a crucial role in cell life [26]. To explore the importance of amino acid addition for cell growth of *C. carboxidivorans* P7, the effects of 20 conventional amino acids on cell growth were studied by serum bottle fermentation in MM520 medium. Fermentation results showed that histidine and aspartic acid promoted cell growth, but the presence of arginine significantly impaired cell growth (Fig. 1A). It was speculated that arginase (EC: 3.5.3.1) probably converted arginine to urea and ornithine, which consequently may have contributed to increase in the metabolic load of cells and decreased cell growth. KEGG analysis revealed that *C. carboxidivorans* P7 lost the ability to yield citrulline from arginine due to the absence of arginine deiminase. We hypothesized that if cells could generate citrulline from arginine, they could then break down arginine through the ADI (arginine deiminase) pathway, hence providing cells with additional ATP supply. Although the complete ADI metabolic pathway does not exist in *C. carboxidivorans* P7, it does have a complete citrulline degradation pathway. Furthermore, it was known that citrulline could be transformed to carbon dioxide, water, ammonia, and two molecules of ATP by carbamoyl phosphate synthetase [27]. Therefore, citrulline was added to explore the effects on the cell density of *C. carboxidivorans* P7. The results indicated that the addition of citrulline significantly promoted the growth capacity of *C. carboxidivorans* P7, and the maximum OD_600_ increased by 18.6% compared with the wild-type strain (Fig. 1A).

To further determine the optimal concentration of citrulline addition, 0.6 g/L, 1.2 g/L, and 2.4 g/L citrulline were added to the fermentation medium and incubated under the same conditions. The results showed that the addition of 0.6 g/L citrulline was most beneficial for cell growth. The stable phase of the cells was obviously prolonged and the sugar consumption was significantly promoted. The glucose was depleted after 24 h fermentation. Increasing the concentration of citrulline further resulted in decreased cell growth compared with the addition of 0.6 g/L citrulline (Fig. 1B and C). It may be due to the limited use of citrulline by *C. carboxidivorans* P7. As shown in Fig. 1D, there were still a large amount of citrulline residues in the later stage of fermentation under the addition of 1.2 g/L or 2.4 g/L citrulline.

### Heterotrophic growth experiments proved that citrulline metabolism promoted cell glycolysis and delayed cell aging

**Fig. 2:**
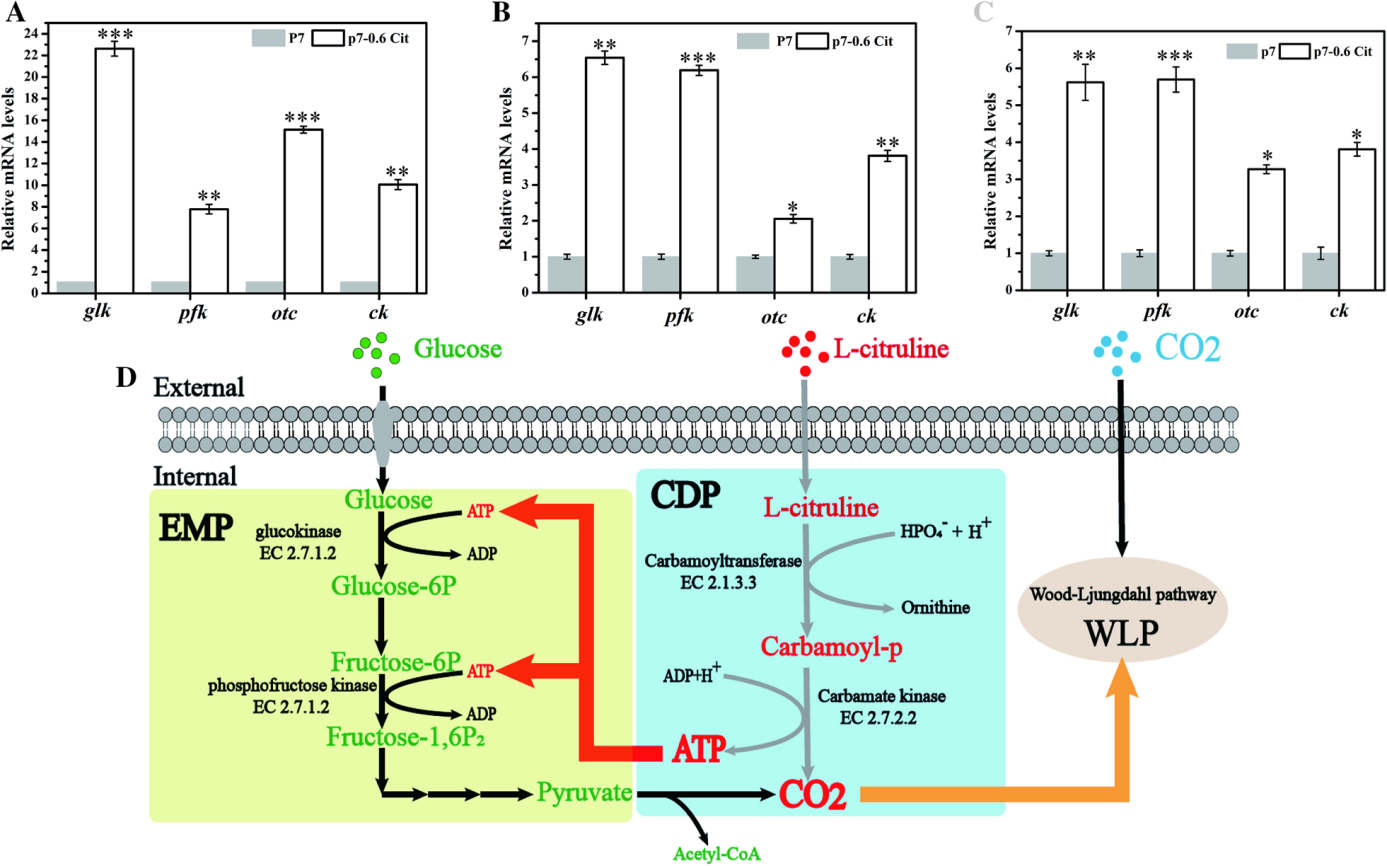
Expression of key genes related to citrulline metabolism pathway and key genes of EMP pathway were analyzed by RT-qPCR during *C. carboxidivorans* P7 heterotrophic fermentation in different medium. RT-qPCR analysis of *C. carboxidivorans* P7 fermented for 12 h (**A**), 24 h (**B**), and 36 h (**C**). **D** Citrulline metabolism pathway. (P7: *C. carboxidivorans* P7 was fermented in MM520 medium. P7-0.6 g/L Cit: *C*. *carboxidivorans* P7 was fermented in MM520-0.6 Cit medium.) *P < 0.05. **P < 0.01. ***P < 0.001

**Fig. 3:**
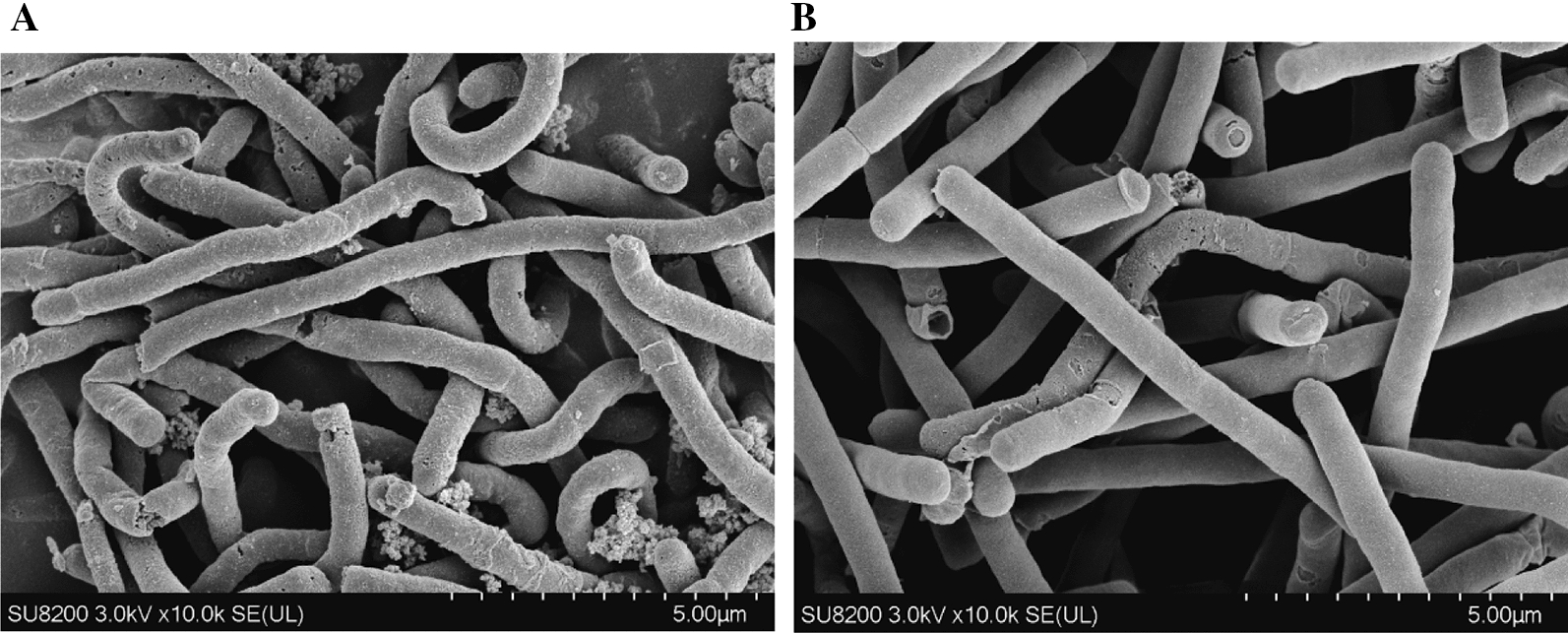
FSEM analysis of *C. carboxidivorans* P7 fermented in MM520 medium (**A**) and MM520-0.6 Cit medium (**B**) for 96 h

Based on the above results, the addition of citrulline boosted cell growth, accelerated glucose utilization, and prolonged cell stability. It is known that citrulline can generate carbamoy-P under the action of ornithine carbamoyltransferase; then the carbamoy-P generates CO_2_ and ATP under the action of carbamate kinase (Fig. 2D). Therefore, it was speculated that citrulline catabolism provided additional ATP supply for glycolysis, thereby improving the ability of two key energy-consuming enzymes (*glk* and *pfk*), in turn increasing glucose utilization. Under the same conditions, *C. carboxidivorans* P7 was fermented in serum bottle using MM520-0.6 Cit and MM520 medium for the same time and samples were taken regularly. RT-qPCR was used to analyze the expression levels of *glk* (encoding glucokinase), *pfk* (encoding phosphofructokinase), *otc* (encoding ornithine carbamoyltransferase), and *ck* (encoding carbamate kinase) at 12 h, 24 h, and 36 h, respectively. The results showed that in MM520-0.6 Cit medium, *glk*, *pfk*, *otc,* and *ck* were significantly up-regulated at 12 h, 24 h, and 36 h (Fig. 2A, B, and C). However, after 24 h of fermentation, due to the consumption of citrulline and glucose, the relative expression of *glk*, *pfk*, *otc*, and *ck* decreased accordingly. After the fermentation at 96 h, it was found that the cells cultured in MM520-0.6 Cit medium had smooth surface and no residue of glucose particles observed by frozen scanning electron microscopy (FSEM) (Fig. 3B). On the contrary, the surface of cells cultured in MM520 medium had a rough surface with more glucose residues (Fig. 3A). It was also reported that cells had obvious differences in cell morphology at different growth stages [12]. In the decline phase, the cell surface would become rough compared with the early phase [12]. So, it was speculated that the cell morphology may be able to reflect the growth state of the cells. As a result, the FSEM experiments also showed that cells with citrulline addition had a more smooth surface (Fig. 3B). So indicated that citrulline promoted cell viability and delayed cell aging. Therefore, FSEM analysis further showed that the addition of citrulline contributed to promote the metabolism of glucose by *C. carboxidivorans* P7 and could maintain cell viability.

### Citrulline boosted heterotrophic growth of *C. carboxidivorans* P7 while supplying extra ATP

**Fig. 4:**
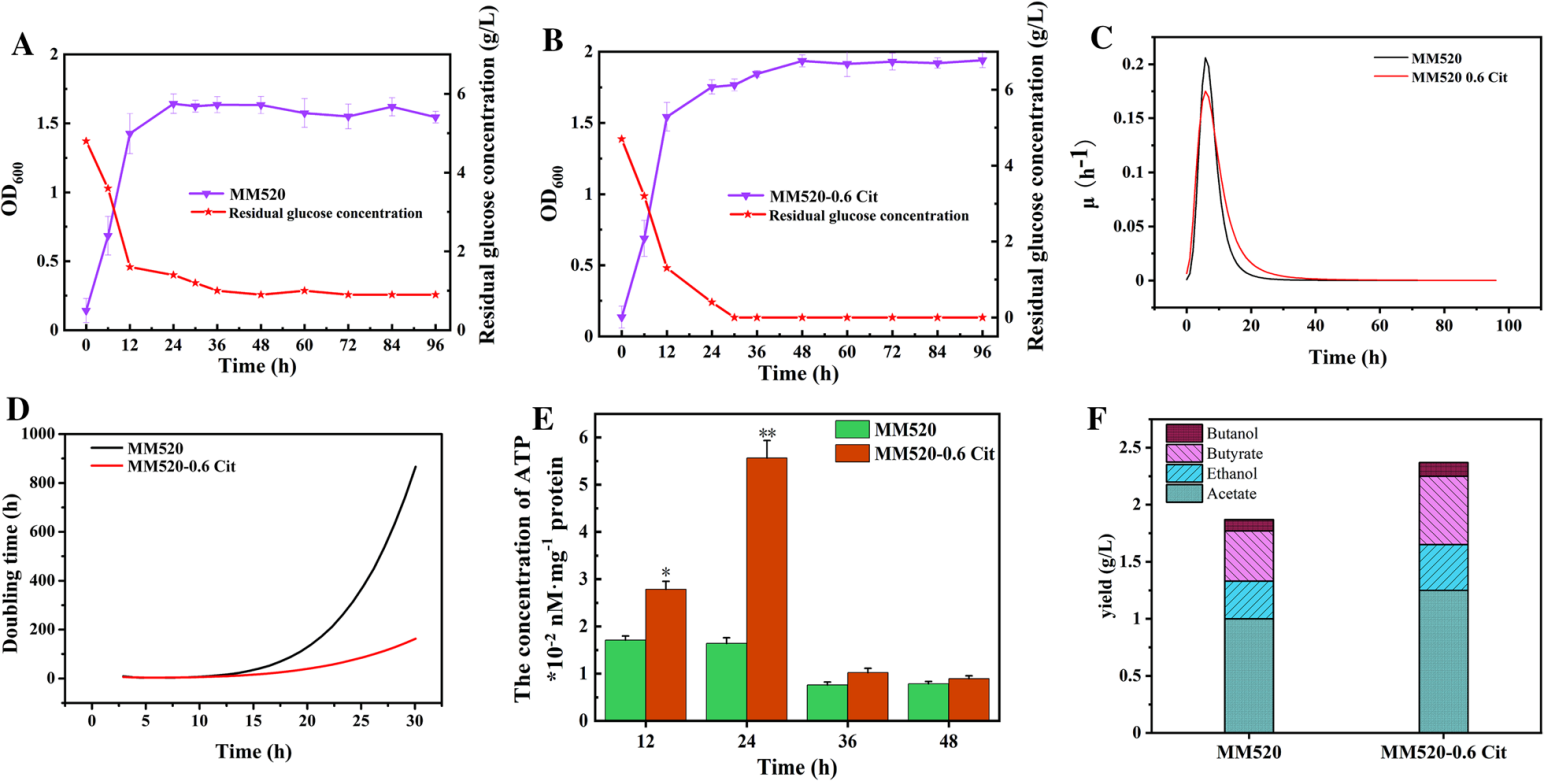
Under heterotrophic conditions, the effects of citrulline on the fermentation of *C. carboxidivorans* P7. *C. carboxidivorans* P7 was cultivated in MM520 medium (**A**) and MM520-0.6 Cit medium (**B**). **C** Doubling time, **D** Specific growth rate, (**E**) The concentration of ATP. **F** The yield of ethanol, butanol, acetate, and butyrate in heterotrophic conditions. *P < 0.05. **P < 0.01

By analyzing the metabolic pathway of citrulline, it was further speculated that citrulline catabolism could provide cells with additional ATP, thereby promoting cell growth. Therefore, under the same conditions, *C. carboxidivorans* P7 was fermented in serum bottles with MM520-0.6 Cit and MM520 medium for the same time. The specific growth rate, doubling time, and intracellular ATP levels of the cells were further analyzed. The results showed that the specific growth rate of *C. carboxidivorans* P7 in MM520-0.6 Cit medium increased significantly when the fermentation was performed within 10 h to 30 h (Fig. 4). It is known that citrulline can be transformed to carbon dioxide, water, ammonia, and two molecules of ATP by carbamoyl phosphate synthetase. What is more, it was also shown that citrulline addition promoted the utilization of glucose (Fig. 1C). As a result, the addition of citrulline provided additional ATP and enhanced glucose consumption. However, the increase in glucose consumption would also produce more acids. So, acids were increased when citrulline was added (Fig. 4F). When fermentation reached 30 h, the cell doubling time of *C. carboxidivorans* P7 in MM520-0.6 Cit medium was 162 h, which was 81.3% shorter than that of MM520 medium. Through analysis of intracellular ATP level, it was found that the ATP level in MM520-0.6 Cit medium was 3.39-fold higher than that in MM520 medium at 24 h. In addition, it was reported that the addition of arginine increased the intracellular energy ATP level by fivefold in *C*. *autoethanogenum* [15]. Metabolic modeling and experiments also showed increased in ATP production through the arginine deiminase pathway [15]. It should be noted that the mechanism of arginine metabolism to promote ATP accumulation is similar as that of citrulline metabolism. Therefore, it was believed that the higher ATP level was contributed with citrulline addition. The results herein showed that the addition of citrulline increased intracellular ATP levels under heterotrophic conditions. Thereafter, when citrulline was depleted, the intracellular ATP level decreased and remained at a lower level.

Under heterotrophic conditions, metabolites of *C. carboxidivorans* P7 in MM520 and MM520-0.6 Cit medium were analyzed (Table 1). The addition of citrulline had no significant effect on product conversion of ethanol and butanol. However, the addition of citrulline could promote the utilization rate of glucose in cells. Although the product conversion rate did not change significantly, the total alcohol yield of fermentation products (the yield of ethanol and butanol was 1.2 g/L and 0.16 g/L, respectively) increased by about 20% (Fig. 4F).

**Table 1:** Under heterotrophic conditions, the product yields of *C. carboxidivorans* P7 under different culture conditions

Medium	Acetate (g/g)	Ethanol (g/g)	Butyrate (g/g)	Butanol (g/g)	Total alcohols (g/g)	Total acid (g/g)	Alcohols/acids (g/g)	C4/C2 (g/g)	C recovery (%)
MM520	0.30 ± 0.01	0.07 ± 0.01	0.15 ± 0.01	0.025 ± 0.002	0.10 ± 0.01	0.46 ± 0.04	0.21	0.47	57
MM520-0.6 Cit	0.29 ± 0.02	0.08 ± 0.01	0.16 ± 0.01	0.024 ± 0.001	0.10 ± 0.01	0.45 ± 0.02	0.22	0.49	56.4

### Citrulline boosted autotrophic growth of *C. carboxidivorans* P7 while supplying extra ATP

**Fig. 5:**
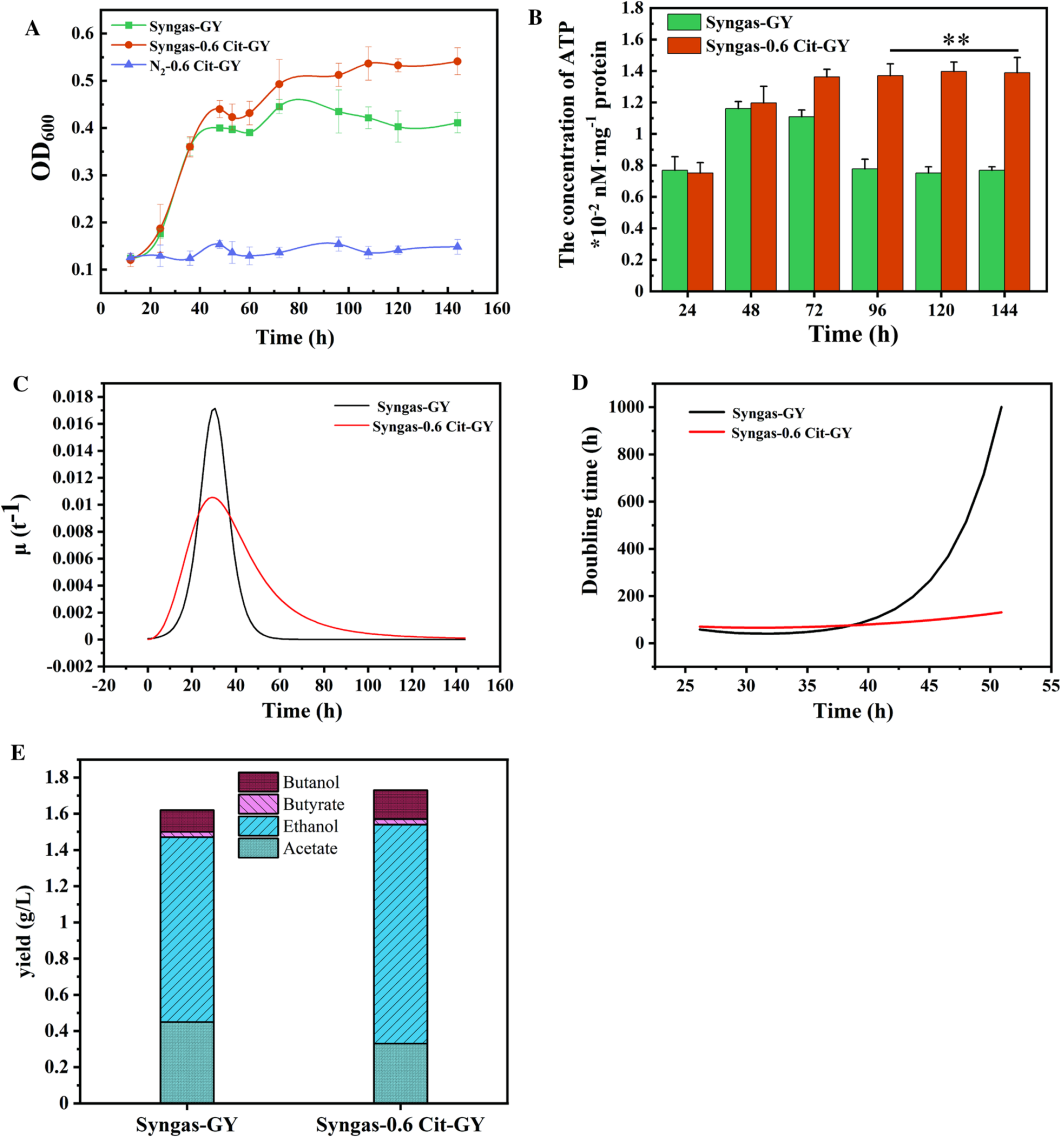
Under autotrophic conditions, the effects of citrulline on the fermentation of *C. carboxidivorans* P7. **A** Cell density, **B** The concentration of ATP, **C** Specific growth rate, **D** Doubling time. **E** The yield of ethanol, butanol, acetate, and butyrate in autotrophic conditions. **P < 0.01

To further verify whether citrulline could provide cells with ATP and promote cell growth under autotrophic conditions (CO_2_/CO/H_2_ [50:35:15], 1 atm), *C. carboxidivorans* P7 was fermented in Syngas-GY free and Syngas-0.6 Cit-GY free medium (pH 6.6, 37 ℃, 150 rpm) and the effects of citrulline improved the growth of *C. carboxidivorans* P7 was investigated. Firstly, as shown in Fig. 5A, when citrulline was added to the N_2_-GY free medium, the cells could not grow normally, indicating that citrulline could not be used as the only carbon source to maintain cell growth. However, under syngas conditions, exogenous addition of citrulline could increase the biomass of *C. carboxidivorans* P7 by 31.6%. It was also found that the ATP levels in Syngas-0.6 Cit-GY free medium were significantly higher than those in Syngas-GY free medium after fermentation for 48 h. Moreover, at 96 h of fermentation, although the ATP levels in Syngas-0.6 Cit-GY free medium remained almost constant, intracellular ATP levels increased 80.5% in Syngas-0.6 Cit-GY free medium compared to Syngas-GY free medium (Fig. 5B). It was also found that the specific growth rate of *C. carboxidivorans* P7 in Syngas-0.6 Cit-GY free medium increased significantly, while the doubling time of the bacteria greatly shortened after the fermentation reached 40 h (Fig. 5C and D).

Under autotrophic conditions, the metabolites of *C. carboxidivorans* P7 were analyzed in Syngas-0.6 Cit-GY free and Syngas-GY free medium (Table 2). The addition of citrulline increased the yield of ethanol and the alcohol/acid ratio by 18.6% and 60.3%, respectively. Although the yield of ethanol reached 1.21 g/L, there was no significant change in butanol production. A possible explanation could be that the low production of butanol in *C. carboxidivorans* P7.

**Table 2:** Under autotrophic conditions, the product yields of *C. carboxidivorans* P7 under different culture conditions

Medium	Acetate (g/L)	Ethanol (g/L)	Butyrate (g/L)	Butanol (g/L)	Total alcohols (g/L)	Total acid (g/L)	Alcohols/Acids (g/g)	C4/C2 (g/g)
Syngas-GY	0.45 ± 0.05	1.02 ± 0.11	0.12 ± 0.03	0.03 ± 0.01	1.05 ± 0.12	0.57 ± 0.08	1.84	0.10
Syngas-0.6 Cit-GY	0.26 ± 0.04	1.21 ± 0.09	0.16 ± 0.02	0.03 ± 0.01	1.24 ± 0.10	0.42 ± 0.06	2.95	0.13

## Discussion

The WLP pathway exists in *C*. *carboxidivorans* P7, which has been found in most acetic bacteria, such as *Acetobacterium woodii* [28], *Clostridium formicoaceticum* [29], *Clostridium aceticum* [30], *Clostridium ljungdahlii* [31], and *C. autoethanogenum* [32]. Conforming to the current mainstream of clean energy, *C. carboxidivorans* P7 is able to produce biofuels from syngas via the WLP pathway. However, *C. carboxidivorans* P7 has low cell density and insufficient intracellular energy supply during the syngas fermentation process, rendering it industrially ineffective. In this study, it was found that the addition of citrulline not only improved cell density but also enhanced the intracellular ATP level in *C*. *carboxidivorans* P7 under both heterotrophic and autotrophic conditions. Moreover, the addition of citrulline increased the yield of ethanol and the alcohol/acid ratio by 18.6% and 60.3%, respectively. Therefore, this research revealed that the exogenous addition of citrulline increased the biomass and ATP supply, which had important implications for accumulating biofuels in *C*. *carboxidivorans* P7.

Recently, cell immobilization technology, optimization of fermentation parameters, and metabolic engineering have been used to improve cell density and intracellular ATP level. Cell recovery [33] and cell immobilization [34] techniques promoted cell density during the period of fermentation, resulting in a fivefold increase in butanol production. The control of fermentation processes, such as pH [20], temperature [35, 36], pressure [20], and inoculation amount [37], also had a great influence on cell density, but was not reported to significantly promote the accumulation of intracellular energy ATP. Amino acids are essential elements for life [38], and amino acid metabolism can produce carbon dioxide and other small molecules for cell life activities [13]. Therefore, addition of 5 g/L arginine [15] not only increased the cell density of *C. autoethanogenum* but also increased the intracellular energy ATP level by fivefold. The engineering modification of strains is also of great significance for improving cell fermentation density and intracellular energy ATP accumulation. Ferredoxin oxidoreductase (AOR) [39] provided extra ATP supply for acetic acid bacteria and promoted the accumulation of ethanol and butanol. *C. autoethanogenum* [16] explored the AOR/ADH pathway to increase the level of intracellular energy ATP and increase the yield of ethanol. Overexpression of two glycerol dehydrogenase (GldH) genes (*dha*D1 and *gld*A1) in *Clostridium pasteurianum* further resulted in a 43% increase in glycerol utilization and a significant increase in cell density (> 50%) [40]. At present, the genetic operation system of *C. carboxidivorans* P7 has been preliminarily established [7]. However, there are some problems, such as complex operation, low transduction efficiency, and time consuming, making it difficult to operate in practice. Therefore, cell immobilization and optimization of fermentation parameters are still the main means to improve cell density and intracellular energy ATP supply.

Although this study achieved a synchronous increase in cell density and intracellular energy ATP of *C. carboxidivorans* P7 by exogenous addition of citrulline, the cell utilization of citrulline was not very high. Furthermore, since the genetic operation system of *C. carboxidivorans* P7 is relatively complex and inefficient, it is possible to overexpress the two key enzymes (ornithine carbamoyltransferase (OTC) and carbamate kinase (CK)) of the citrulline pathway in *C. carboxidivorans* P7, to expand the pathway of citrulline metabolism and further improve citrulline utilization of *C. carboxidivorans* P7.

## Conclusions

In general, citrulline could promote the accumulation of intracellular energy ATP, increase the specific growth rate, and shorten the doubling time of *C. carboxidivorans* P7 under both autotrophic and heterotrophic conditions. Under heterotrophic conditions, the addition of citrulline increased the level of intracellular ATP and the production of total alcohol by 3.39-fold and 20%, respectively. Under autotrophic conditions, the addition of citrulline increased the level of intracellular ATP by 80.5%, the yield of ethanol by 18.6%, and the alcohol/acid ratio by 60.3%. In summary, this is the first report to reveal that citrulline could promote the growth of *C. carboxidivorans* P7 and increase intracellular energy ATP, which is of great significance for improving the production of clean energy, such as ethanol and butanol by *C. carboxidivorans* P7.

## Methods

### Strains and culture medium

The strains and primers in the work are summarized in Table 3. *C. carboxidivorans* P7 (DSM 15243) was preserved at − 80 ℃ in our laboratory. It was inoculated into the MM520 medium, containing K_2_HPO_4_ 2.2 g/L, KH_2_PO_4_ 1.5 g/L, (NH_4_)_2_SO_4_ 1.3 g/L, yeast extract 2 g/L, tryptone 4 g/L, Na-Resazurin solution 0.1% (w/v), L-cysteine-HCl∙H_2_O 0.5 g/L, FeSO_4_∙7H_2_O 0.00125 g/L, CaCl∙2H_2_O 0.075 g/L, MgCl_2_∙6H_2_O 0.2 g/L, and trace element solution (FeCl_2_∙4H_2_0 1.5 mg/L, ZnCl_2_ 0.07 mg/L, MnCl_2_∙4H_2_0 0.1 mg/L, CoCl_2_∙6H_2_O 0.19 mg/L, CuCl_2_∙2H_2_O 0.002 mg/L, NiCl_2_∙6H_2_O 0.024 mg/L, H_3_BO_3_ 0.006 mg/L, Na_2_MoO_4_∙2H_2_O 0.036 mg/L). Dissolved oxygen in the medium was boiled for half an hour to eliminate under the stream of N_2_ gas, then added L-cysteine-HCl∙H_2_O 0.5 g/L and continued to boil for an hour until the color of the medium from blue to colorless. The boiled medium was divided into a serum bottle (50 mL/100 mL) and nitrogen added for 10 to 20 min, until the color changed from a light blue to yellow. Then serum bottles were sealed with gas impermeable butyl rubber septum-type stoppers and aluminum crimp seals. The serum bottles filled with medium were sterilized at 121 ℃ for 20 min. 500 μL sterilized glucose (500 g/L) and 250 μL sterilized L-cysteine-HCl∙H_2_O (100 g/L) were added into media. Before inoculation, the culture medium should be adjusted to pH ~ 6.6 with 1 M HCl or 1 M NaOH, which was purged with N_2_ for 10 min and sterilized. The gas fermentation medium (Syngas-GY free medium): MM520 medium contains no glucose and yeast extract and is filled with the gas mixture (50% CO_2_, 35% CO, and 15% H_2_ at 1 atm) and it should be purged every 48 h. The gas fermentation medium (N2-GY free medium): MM520 medium filled with the N_2_ and purged every 2 days, without any glucose and yeast extract added to it.

**Table 3:** Strains and primers in this study

	Characteristics	Source
Strains		
*C. carboxidivorans* P7	Autotrophic growth on CO, CO_2,_ and H_2_	Our lab
Primers 5′ → 3′		
RT-PCR-*glk* F	AGGTACGTGATAAAGCAT	This study
RT-PCR-*glk* R	TGAAACTCCTCCTCCAAT	This study
RT-PCR-*pfk* F	TCCTGAGAAAGGCTACAA	This study
RT-PCR-*pfk* R	ATCTGCTCCACCAATACC	This study
RT-PCR-*otc* F	GAAGAATGGGAAGAACG	This study
RT-PCR-*otc R*	CTGGCAAGCAATGAAGA	This study
RT-PCR-*ck* F	TGAGAAAGGCACTGGAT	This study
RT-PCR-*ck* R	TATGGGTTTAGTTGGAT	This study

### Fermentation

All fermentation processes were performed in serum bottles filled with 50 mL culture medium, and samples were taken at regular intervals for analysis. In order to study the effects of 20 conventional amino acids and citrulline on the growth of *C. carboxidivorans* P7, each amino acid (1 g/L) was added to the MM520 medium and placed in a 37 ℃ incubator for fermentation. So as to further study the effects of different concentrations of citrulline on the growth of *C. carboxidivorans* P7, different concentrations of citrulline (0.6 g/L, 1.2 g/L, 2.4 g/L) were added to the fermentation medium. Heterotrophic medium supplemented with 0.6 g/L citrulline was named MM520-0.6 Cit. Syngas fermentation was performed in Syngas-GY free medium. After inoculation at 5% (v/v), the serum bottle cells were cultured at 37 ℃, 150 rpm. Syngas-GY free medium supplemented with 0.6 g/L citrulline named Syngas-0.6 Cit-GY free medium. All fermentation conditions were performed in duplicate.

### Analytical methods

The spectrophotometer (UV-1800) was used to determine the Optical Density (OD_600_). The specific growth rate (μ) was measured according to derivative of growth curve of *C*. *carboxidivorans* P7. Double time was measured based on the formula (μ = ln2/td, where μ is specific growth rate and td is double time). Acetate and butyrate were analyzed on an HPX-87H column (Bio-Rad). Detection conditions: Temperature 50℃, Mobile phase: 5 mm H_2_SO_4_, Detection wavelength: 210 nm, Flow rate: 1 mL/min. Ethanol and butanol were detected according to Cheng [7]. ATP was measured by ATP Assay Kitae (s0026b) purchased from Beyotime Institute of Biotechnology. All of the assays were performed in triplicate.

### Gene expression detection via RT-qPCR

The total RNA extraction of the collected cells was performed by using FastPure Cell/Tissue Total RNA Isolation Kit RC101(Vazyme Biotech Co., Ltd., Nanjing, China). RT-qPCR reactions were conducted with ChamQ Universal SYBR qPCR Master Mix*Q711-02 (Vazyme Biotech Co., Ltd., Nanjing, China). The StepOnePlus 96 real-time PCR system (Applied biological systems Inc, USA) was used to amplify and quantify the PCR samples. The method was as follows: 30 s at 95 ℃, 40 amplification cycles of 10 s at 95 ℃, and 30 s at 60 ℃. Relative levels of transcript abundance of the studied genes were calculated by the 2^−∆∆CT^ method. RT-qPCR of each gene was tested with three reactions in parallel.

### Scanning electron microscopy

Frozen scanning electron microscopy (FSEM) was used to observe cell morphology of *C. carboxidivorans* P7 under the medium of MM502 and MM520-0.6 Cit. Bacterial cells were cultured in MM520 and MM520-0.6 Cit medium at 37℃ incubator for 96 h. Then the bacteria cells were collected by centrifugation at 6000 rpm, resuspended with sterile water, and placed on carbon film-coated copper Grid (230 mesh; Beijing Zhongjing Science and Technology Co., Ltd., Beijing, China). At last, the bacterial cells liquid on the film was dried at 25 ℃ and observed by FSEM.

## Data Availability

The datasets used and/or analyzed during the current study are available from the corresponding author on reasonable request.
